# The social physics collective

**DOI:** 10.1038/s41598-019-53300-4

**Published:** 2019-11-12

**Authors:** Matjaž Perc

**Affiliations:** 1 Faculty of Natural Sciences and Mathematics, University of Maribor, Koroška cesta 160, 2000 Maribor, Slovenia; 2 Department of Medical Research, China Medical University Hospital, China Medical University, Taichung, Taiwan; 3 Complexity Science Hub Vienna, Josefstädterstraße 39, 1080 Vienna, Austria

**Keywords:** Complex networks, Statistical physics, Social evolution, Climate-change mitigation

## Abstract

More than two centuries ago Henri de Saint-Simon envisaged physical laws to describe human societies. Driven by advances in statistical physics, network science, data analysis, and information technology, this vision is becoming a reality. Many of the grandest challenges of our time are of a societal nature, and methods of physics are increasingly playing a central role in improving our understanding of these challenges, and helping us to find innovative solutions. The Social physics Collection at *Scientific Reports* is dedicated to this research.

Although we are unique and hardly predictable as individuals, research has shown that in a collective we often behave no differently than particles in matter^1^. Indeed, many aspects of collective behavior in human societies have turned out to be remarkably predictable, and this fact has paved the way for methods of physics to be applied to many contemporary societal challenges. Examples include traffic^2^, crime^3^, epidemic processes^4^, vaccination^5^, cooperation^6^, climate inaction^7^, as well as antibiotic overuse^8^ and moral behavior^9^, to name just some examples.

In fact, possible synergies between physical and social sciences have been floating around in the scientific literature for centuries. Over two centuries ago, the French political and economic theorist Henri de Saint-Simon was amongst the first to propose that society could be described by laws similar to those in physics^10^. However, similar ideas have been around already in the 17th century, when Thomas Hobbes based his theory of the state on the laws of motion, in particular on the principle of inertia, which was then deduced by his contemporary Galileo Galilei^11^. The ‘invisible hand’ proposed by Adam Smith in the second half of the 18th century is also eerily similar to the now famous notions of economic and social self-organization^12,13^, and at the time was deemed to be as dependable in operation as the law of gravity^14^. And in the 19th century, the evolving physical theories of matter as a vast collection of atoms and molecules inspired a statistical view of societies and the predictable averages therein. Just as the random movements of molecules in a gas yield the mathematically simple gas laws, it was fathomed that societies may also be predictable in the collective scale. Thus, as Philip Ball argued aptly^10^, early sociology was indeed constructed according to an unspoken faith that there was a kind of ‘physics of society’.

But despite the long and fascinating history, it was not before the very end of the 20th century that truly remarkable progress had begun along the interface of physical and social sciences. This progress has been driven by advances in statistical and theoretical physics, by the coming of age of network science^15^ and computational social science^16^, and by the relentless innovations in computer and information technology. The result today is social physics, or the physics of social systems, which is rapidly gaining momentum and developing into a research tour de force for a better tomorrow.

The Social physics Collection at *Scientific Reports* is dedicated to this line of research, and after only half a year in the making underlines its strong potential. Given the diversity of the topics that are covered by social physics, it is challenging to pull a common thread through, and even to select, contributions that have been published thus far in a brief editorial. Therefore, in what follows, only a few representative examples are highlighted.

Wachs and Kertész^17^ present a network-based framework to detect cartels – groups of firms that set prices collectively to increase profits at the expense of consumers – based on the interaction patterns between them. They apply their method to a school milk market in the Republic of Georgia that consists of nearly 150,000 public contracts, revealing groups of firms with high cohesion and exclusivity that are significantly more likely to display cartel behavior.

Martinez-Vaquero *et al*.^18^ study the dynamics of recruitment into organized crime and terrorist networks, using evolutionary game theory as the backbone. Their research identifies key factors that influence the growth and decline of such criminal structures, and it reveals the convoluted interplay between agents that associate with illicit groups, criminals that act on their own, and the rest of the civil society.

De Nadai *et al*.^19^ study whether human cognition imposes constraints in the digital space similar to those that we know exist in the physical space, such as keeping the number of friends and favorite places stable over time. Indeed this seems to be the case, with the results painting an intriguing picture that links human behavior in the physical and digital worlds, bridging research across computer science, social physics, and computational social science.

In addition to the three examples above, several other fascinating papers have been published recently, for example by Santos *et al*.^20^ studying the role of reward and punishment in climate change dilemmas, by Johnson *et al*.^21^ studying the emergent dynamics of extremes in a population driven by common information sources and new social media algorithms, and by Agarwal *et al*.^22^ studying the identification and characterization of teleconnections on different scales through networks. There is no doubt that the Social physics Collection will amass a number of fascinating contributions over time. We encourage the readers to stay tuned, and we cordially invite prospective authors to consider contributing to the Collection.

With all the current and upcoming scientific and technological breakthroughs, it is easy to be excited about what the future holds. But there are formidable challenges up ahead as well. Examples include climate inaction, the depletion of natural resources, growing inequality, and perpetual conflicts in some parts of the world. These challenges, although primarily of a societal nature, require insights from different fields of research to be solved successfully. And physics has in recent years emerged as an important piece of this puzzle.

In the 20th century, physics gave us, among many other great discoveries, the atomic bomb. And although it would seem that peace and prosperity can sometimes benefit from such a show of strength, the hope is that the physics of the 21st century will give us a better understanding of our societies so that the universal goals of humanity can be achieved without force. If social physics will ultimately stand for that, all goals of this line of research would be achieved.
